# Impact of COVID-19 pandemic on relapse of individuals with severe mental illness and their caregiver's burden

**DOI:** 10.3389/fpubh.2023.1086905

**Published:** 2023-02-01

**Authors:** Sara Nooraeen, Shahrzad Bazargan-Hejazi, Morteza Naserbakht, Camelia Vahidi, Farideh Shojaerad, Seyedeh Sahar Mousavi, Seyed Kazem Malakouti

**Affiliations:** ^1^Mental Health Research Center, School of Behavioral Sciences and Mental Health, Iran University of Medical Sciences, Tehran, Iran; ^2^Department of Psychiatry, Charles R. Drew University of Medicine and Science, Los Angeles, CA, United States; ^3^Mental Health Research Center, Psychosocial Health Research Institute, Tehran, Iran; ^4^Andisheh-Salamat-Ravan Mental Rehabilitation Center, Tehran, Iran

**Keywords:** SMIs, COVID-19, caregiver burden, telepsychiatry, Iran, schizophrenia, bipolar mood disorder

## Abstract

**Background:**

The implementation of quarantine and social distancing measures to control the COVID-19 pandemic led to restrictions at the community level and most of in-person psychiatric services were discontinued. This situation could affect the psychopathology of the patients and the burden of their caregivers. The aim of this study was to investigate the effects of COVID-19 pandemic on people with severe mental illnesses (SMIs) and their caregivers' burden.

**Method:**

The study sample consisted of 86 patients with severe mental illness and 86 caregivers. The mental status, relapse rate, and rehospitalization rate of the patients and the general health status and burden of caregivers were investigated in three waves, including before and 3 and 6 months after the COVID-19 pandemic.

**Results:**

The relapse rate of the patients was 14%, 33.7%, and 43% (*p* = 0.000) and the rehospitalization rate was 4.7%, 7%, and 10.5% in waves 0, 1, and 2, respectively (*p* = 0.000). Most of the psychopathological scales increased in three waves (*p* = 0.000). The caregivers' burden and health condition worsened during the nine months of the study as well (*p* = 0.000).

**Conclusion:**

The COVID-19 pandemic led to the exacerbation of symptoms and increased the relapse rate in people with SMIs. It also worsened the caregivers' condition. People with severe mental illnesses (SMIs) and their caregivers are one of the most vulnerable groups on which the COVID-19 pandemic had a marked negative effect.

## Introduction

According to Lancet commission report convened by the world experts in psychiatry in 2018, the universal increase in mental disorders will cost the global economy $16 trillion by 2030 (1). In 2019, the global outbreak of the novel coronavirus (COVID-19) added another layer of concern to the emerging public mental health crisis (2) due to the implementation of lockdown, social distancing, isolation, and quarantine measures to limit its spread (3–5). In the general population, COVID-related restrictions have led to lifestyle disruption, job loss, sleep disturbances, anxiety, depression, and PTSD (6–9). In patients exposed to COVID-19, these restrictions could be exacerbated by fear of isolation, loneliness and boredom, affecting their mental health and even triggering suicidal ideation and suicide (10–12). The pandemic has also prevented people from properly mourning for their beloved ones who were lost to the disease (13). Studies that were conducted during the COVID-19 pandemic found that mental health of the participants significantly decreased during this time compared to the pre- pandemic years (5, 14, 15). The combination of these factors has turned COVID-19 into a crisis in terms of mental health among others. The SARS pandemic and its impact on the needs of patients with pre-existing psychiatric disorders suggest that the health consequences of the pandemic on this vulnerable population could be profound (16, 17). Patients with severe mental illness (SMI) such as schizophrenia or bipolar I disorder often receive a variety of treatments including pharmacological treatment, psychosomatic treatment, and rehabilitation (18). Any disruption in their routine care, as a result of pandemic lockdown policies, is likely to exacerbate their conditions (19, 20).

It has been reported that interruptions in the mental health-related utilization of SMIs are associated with a higher risk of recurrence and relapse of symptoms and readmission to the psychiatric ward in these patients (21–23). Furthermore, the job loss rate has been higher for those with psychiatric diseases at the time of the pandemic compared to the general population (24). This further increases the vulnerability of this group which may receive less attention in the pandemic situation compared to the general population (8, 9, 14, 15, 25–28).

The caregivers of SMI patients play a major role in the management of their patients. Whether caregiving is provided by a family member or a formal caregiver, it is part of the core care system that assists patients in getting prescribed treatments and ensures the continuity of care (29). It is common for caregivers to feel frustrated, stressed, and helpless while trying to strike a balance between the responsibilities of the role and providing the best care without burdening their health (30–32). A review of the literature regarding the caregivers' experiences shows a prevalence of 14–47% for depression and anxiety among the caregivers of the SMI patients (33). In the meantime, a higher prevalence is reported for caregivers of schizophrenia patients (30%) (34, 35). Additionally, the caregivers of the SMI patients have lower levels of perceived social support and quality of life (29). The existing evidence supports the role of community mental health programs in enhancing the quality of life of both caregivers and their SMI patients (36–38). However, the impact of the closure of community-based mental health centers due to the COVID-19 pandemic on the mental health condition of Iranian SMI patients is unclear. The aim of the present study was to estimate the impact of the COVID-19 pandemic on the psychopathology of individuals with SMIs and their caregivers' perceived burden.

## Methods

### Study design and participants

A cohort study was conducted between August 2020 and December 2021. The participants were recruited from the Andishe Salamat Ravan (ASR), Tehran, Iran. ASR is a community-based day-care rehabilitation center that provides mental rehabilitation and outreach services for chronic psychiatric patients. The patients were eligible to participate if they were above 18 years, had a diagnosis of SMI including schizophrenia, bipolar disorder, and chronic major depressive disorder, and were receiving regular care from the ASR (before the pandemic). The eligibility criteria for the caregivers were age above 18 years. The participants that provided written informed consent were scheduled for a face-to-face interview at the ASR. They included 86 SMI patients and their caregivers (n = 86). Before the COVID-19 pandemic, as part of the standard of care, these patients were receiving community-based psychiatric services including monthly in-person visits as well as medical and rehabilitation care in the center 3–4 days per week. However, these services were disrupted during the COVID-19 pandemic due to the implementation of quarantine and social distancing protocols and were replaced by telepsychiatry for patients who had access to the Internet and smartphones. Otherwise, the patients were contacted by phone for counseling and were advised to adhere to their individualized treatment plan. All patients received their medications by mail to avoid possible exposure in the drugstore.

### Data collection and study measures

Data were collected by three trained interviewers: a physician and two psychologists. The interview time was about 30 min for each patient and his/her caregiver.

Data collection took place in three waves: wave zero refers to 6 months before the COVID-19 pandemic. Waves 1 and 2 refer to 3 and 9 months after World Health Organization declared COVID-19 a pandemic on March 11, 2020 (39). The following measures were used to assess the mental health conditions of the patients with SMIs.

#### Positive and negative syndrome scale

The Positive and Negative Syndrome Scale (PANSS) is used to measure the severity of schizophrenia symptoms. It was published in 1987 by Stanley et al. (29). It is known as a gold standard measure for the evaluation of the severity of schizophrenia symptoms. It includes a positive scale (7 items), a negative scale (7 items), and a general psychopathology scale (16 items), and takes about 45 min to complete. The Cronbach's alpha coefficient of the PANSS is 0.77. This scale was used to determine the severity of schizophrenia symptoms before and after the COVID-19 pandemic. The reliability and validity of the Persian version of this instrument were confirmed by Ghamari et al. (40). Diagnostic and clinical researchers have reported that this questionnaire has an acceptable construct validity (40).

#### The young mania rating scale

The YMRS is an 11-item interviewer rated scale (41). The items have five defined grades of severity. Four items are double weighted (irritability, speech, thought content, and disruptive/aggressive behavior (42). This questionnaire was validated by Barekatain et al. in Iran (43). The results of differentiation analysis showed a cut-off point of 17.14, a sensitivity of 98.4%, and a specificity of 98.4%. This scale was used to measure mania symptoms in patients with bipolar disorder.

#### The beck depression inventory

The BDI (44) is a 21-question multiple-choice self-report inventory for measuring the severity of depression. This instrument was validated in Iran in various studies including a study by Ghassemzadeh et al. (45). The BDI was used to evaluate depressive symptoms in patients with bipolar and major depressive disorders.

#### Relapse

Disease relapse was assessed in terms of its significance and rehospitalization. A relapse was “mild” if the severity of the illness and the symptoms were serious enough for the therapist to increase medications and frequency of virtual visits to control the exacerbated symptoms.

Rehospitalization indicated that the severity of the exacerbated symptoms made it impossible to control the symptoms at home. To prevent further harm to the patients and their families, hospitalization was inevitable.

The following measures were used to assess the mental health conditions of the caregivers:

#### The general health questionnaire

The GHQ is a screening tool for identification of minor psychiatric disorders in the general population or within a community or non-psychiatric clinical setting such as a primary care or general outpatient center. The reliability and validity of this questionnaire were evaluated by Taghavi in Iran (46). The coefficients were calculated using three different methods: test-retest, split-half, and Cronbach alpha, which were 0.70, 0.93, and 0.90, respectively. The validity of the questionnaire measured by the Middlesex Hospital Questionnaire (MHQ) was 55 (*P* < 0001). The subscale-total correlations, as another index of validity, were between 0.72 and 0.87 (46). This questionnaire was used to screen the mental health situation of the caregivers.

#### Family burden interview scale

The FBIS (47) is a semi-structural interview measurement tool with a reliability coefficient of 0.72. This scale was used to measure the burden of caregiving. This scale was used by Chimeh et al. in Iran (48).

### Ethical considerations

The protocol of the study was approval by the Ethics Committee of Iran University of Medical Sciences (ethics code: IR.IUMS.REC.1399.416). Written consent was obtained from all participants prior to the study.

### Statistical analysis

Analyses were conducted using IBM SPSS 25 statistical software. Descriptive analyses were used to describe the demographic characteristics of the patients and their caregivers. Mauchly's sphericity test was performed to evaluate the sphericity of the tests. Greenhouse–Geisser correction was applied as an alternative to correct the violation of the sphericity. Repeated Measure ANOVA followed by *post hoc* Bonferroni test was applied to detect any overall difference in the severity of the psychopathological symptoms between the three waves.

Interrater reliability of the interviewers was evaluated using the Pearson correlation coefficient. Statistical significance was determined as *p*-value < 0.05.

## Results

### Demographic profile

Of 86 patients, 11 withdrew from the study [seven patients with a diagnosis of schizophrenia and four with bipolar mood disorder (BMD)]; therefore, the final sample included 75 patients and 75 caregivers. The Mauchly's sphericity test results were not significant for YMRS (*P* = 0.188), BDI (0.070) and RELAPS (Sig = 0.348); therefore, the sphericity assumption was met. However, the sphericity assumption was not met for PANSS (*P* < 0.001), GHQ (*P* < 0.001), FBIS (*P* < 0.001) and rehospitalization (*P* = 0.026). The mean age of the patients was 43.4 ± 9.5 years (range: 26–72 years), and 86.7% were diagnosed with schizophrenia, 86.7% with BMD, and 13.3% with major depressive disorder (MDD). Table 1 shows the demographic characteristics of the patients and their caregivers.

**Table 1:** Sociodemographic characteristics of participants (patients and their caregivers).

		**No**.	**%**
**Patient (*****n** =* **75)**			
**Gender**			
	Male	56	74.70%
	Female	19	25.30%
**Diagnosis**			
	Schizophrenia	65	86.70%
	BMD	8	10.70%
	MMD	2	2.60%
**Education**			
	Illiterate	4	5%
	Middle school	42	56%
	High school diploma	25	34%
	University or higher	4	5%
**Marital status**			
	Single	62	82.70%
	Married	13	17.30%
**Caregiver (*****n** **=*** **75)**			
**Gender**			
	Male	25	33.30%
	Female	50	66.70%
**Relationship to patient**			
	Parents	40	53.40%
	Sibling	24	32%
	Spouse	7	9.30%
	Other	4	5.30%
**Education**			
	Illiterate	35	46.70%
	Middle school	29	38.70%
	High school diploma	9	12%
	University or higher	2	2.60%
**Marital status**			
	Single	21	28%
	Married	54	72%
Mental Illness		19	25.30%
Physical Illness		23	30.70%

### Severity of psychopathology of patients and caregivers in three waves

As demonstrated in Table 2, the patients' mean scores for psychopathology measures increased over the three waves except for YMRS. The relapse rate was 14, 33.7, and 43%, and the rehospitalization rates were 4.7, 7, and 10.5% in waves 0, 1, and 2, respectively (Table 2).

**Table 2:** Repeated measures analysis of variance (ANOVA) for psychopathology of patients and mental health of caregivers.

	**Wave zero**		**Wave 1**		**Wave 2**		**df**	***F***	***P*-value**
	***M***	***SD***	***M***	***SD***	***M***	***SD***			
**Patient**									
PANSS^*^	52.14	15.45	55.39	17.43	73.81	22.11	1.197	69.881	< 0.001
YMRS^**^	7.08	7.26	7.54	5.71	4.30	10.04	2.000	0.440	0.660
BDI^**^	21.00	14.85	25.53	16.74	31.18	12.89	2.000	7.680	0.011
Relapse^**^	0.19	0.497	0.48	0.627	0.64	0.667	2.000	14.099	< 0.001
Rehospitalization^*^	0.05	0.212	0.07	0.256	0.10	0.308	1.846	1.119	0.326
**Caregiver**									
GHQ^*^	15.01	7.11	18.29	9.73	26.68	12.10	1.044	39.047	< 0.001
FBIS^*^	5.57	3.28	8.10	4.00	9.05	3.34	1.009	91.177	< 0.001

PANSS, Positive and Negative Syndrome Scale; YMRS, Young Mania Rating Scale; BECK, Beck Depression Inventory; GHQ, General Health Questionnaire; FBIS, Family Burden Interview Scale 1; ^*^Greenhouse–Geisser; ^**^Wilks' Lambda.

The results of *post hoc* analysis of the severity of psychopathology and relapse rate are presented in Table 3.

**Table 3:** Effect of time passing on psychopathology.

	**WAVE**		**Mean difference**	**Std. error**	***P*-value**	**95% Confidence interval**	
**Patient**							
PANSS	0	1	−3.406	0.885	< 0.001	−5.582	−1.230
	0	2	22.688	2.494	< 0.001	28.823	16.552
	1	2	−19.281	2.418	< 0.001	−25.229	13.334
YMRS	0	1	−2.667	2.682	NS	−10.756	5.422
	0	2	0.889	3.195	NS	−8.746	10.524
	1	2	3.556	4.46	NS	−9.895	17.006
BDI	0	1	−6.182	2.311	NS	−12.815	0.452
	0	2	−15.818	4.486	0.016	−28.693	−2.943
	1	2	−9.636	4.716	NS	−23.172	3.899
Relapse	0	1	−0.291	0.077	< 0.001	−0.48	−0.102
	0	2	−0.453	0.089	< 0.001	−0.67	−0.237
	1	2	−0.163	0.086	NS	−0.372	0.047
Rehospitalization	0	1	−0.023	0.033	NS	−0.104	0.057
	0	2	−0.058	0.042	NS	−0.16	0.044
	1	2	−0.035	0.042	NS	−0.137	0.068
**Caregiver**							
GHQ	0	1	−3.279	0.568	< 0.001	−4.667	−1.892
	0	2	−22.326	3.348	< 0.001	−30.501	14.15
	1	2	19.047	3.287	< 0.001	−27.073	−11.02
FBIS	0	1	−2.535	0.25	< 0.001	−3.144	−1.925
	0	2	−31.767	3.214	< 0.001	−39.617	−23.918
	1	2	−29.233	3.181	< 0.001	−37.002	−21.463

PANSS, Positive and Negative Syndrome Scale; YMRS, Young Mania Rating Scale; BECK, Beck Depression Inventory; GHQ, General Health Questionnaire; FBIS, Family Burden Interview Scale 1.

## Discussion

### Patients

The findings showed that the relapse rate rose from 9.3 to 43% during three waves of the study. Additionally, the patients' symptoms deteriorated 9 months after the COVID-19 pandemic. Similar findings are reported from other countries (24, 49–51). The treatment and management of SMIs such as schizophrenia and bipolar disorders are very costly (52–54). Any increase in the incidence of relapse, as reported in the present study, would not be cost-effective (55). Several researchers have suggested the use of telepsychiatry services as an alternative mitigating strategy to minimize disruption in patient care (56–58). The efficacy of telepsychiatry has been proven in neurotic psychiatric disorders (7, 59, 60). A cross-sectional study conducted in the US found that psychiatric visits to patients through telepsychiatry, mostly by telephone, were much higher compared to before the pandemic and face-to-face visits (61). Studies have shown the efficacy of telepsychiatry services for individuals with SMIs (62, 63). However, in low-to-middle income countries (LMICs), such as Iran, barriers such as lack of access to smartphones, digital illiteracy, poor Internet connections, low telepsychiatry awareness, lack of provider training, and ban of e-prescriptions limit the use of telepsychiatry (64). Therefore, the efficacy and effectiveness of telepsychiatry in LMICs that are disproportionally affected by mental health disorders need further research (65–67).

### Caregivers

According to the findings, caregivers experienced a higher burden and worsening of mental health situation. In the present study, more than 50% of the caregivers were patients' parents. They were mostly old, had low education levels, and had some physical and mental problems, which made it difficult to take care of the patients.

The caregivers of psychiatric patients are a vulnerable group (68) that is sometimes neglected while the condition of the patients can affect them. Findings indicate that caregivers are more likely to have psychological problems in comparison with the general population (69, 70). It could be due to the burden of long-term caring for individuals with chronic mental conditions such as medication costs, cigarette smoking, patient's unemployment, and some subjective reasons like stigma, shame, avoiding friends, etc. All of the above studies were conducted in non-COVID situations (38). Few studies have examined the status of the patients in such pandemic conditions and concluded that the burden of caregivers increased during the COVID-19 pandemic markedly (71, 72). There are several possible reasons for this finding. First, many patients and their caregivers lost their jobs during the pandemic, and the lack of adequate financial and social support has created many financial problems for them, which may lead to an increase in the burden. Second, patients spent several hours outside the home to receive day center services. Third, the patients and families became bored and domestic violence increased (73–75). Fear of getting infected with COVID-19 and concerns about the person who should care for the patient in case of disease or death were also sources of stress for families. In the present study, the relapse rate was rather high in wave two (about 40%); however, few patients were hospitalized, which could be due to the fear of families who do not wish to hospitalize their patients even in the case of severe relapses. Caregivers accepted the responsibility of caring for the patient at home, which also increased their burden in turn. Providing telepsychiatric and telerehabilitation services to patients and their caregivers could be crucial (76). It is predicted (77) that as the pandemic continues, it will be harder for these families to cope, particularly considering the low vaccination rate.

## Limitation

A small sample size and other methodological problems like the lack of a control group could be considered as the study limitations. Although all of the patients received telephone follow-up (since not all of them had smartphone) and receive their medications, it was not possible to assess the impact of telepsychiatry services due to the lack of a control group.

To the best of our knowledge, this is the first study to understand the degree to which the COVID-19 pandemic exacerbated the symptoms in patients with SMIs and the burden of their caregivers in Iran.

## Conclusion

People with SMIs and their caregivers are a vulnerable group during pandemics that may experience the exacerbation of their mental disease. It may also impose more objective and subjective burdens on their families and caregivers (77). They require more attention to keep up with the general population.

## Data availability statement

The original contributions presented in the study are included in the article/Supplementary material, further inquiries can be directed to the corresponding author.

## Ethics statement

The studies involving human participants were reviewed and approved by Ethics Committee of Iran University of Medical Sciences (Ethics code: IR.IUMS.REC.1399.416). The patients/participants provided their written informed consent to participate in this study.

## Author contributions

SN: study design, data gathering, interviewing, and writing manuscript. SB-H: data gathering and writing manuscript. MN: data gathering and data analysis. CV: study design and data gathering. FS and SSM: data gathering and interviewing. SKM: study design and data analysis. All authors contributed to the article and approved the submitted version.
